# Targeting Nectin-4 sensitizes glioblastoma to temozolomide

**DOI:** 10.3389/fphar.2026.1908590

**Published:** 2026-09-08

**Authors:** Xiao-Guang Zhang, Hua Wang, Jin-Ning Song

**Affiliations:** 1 Department of Neurosurgery, The First Affiliated Hospital of Xi’an Jiaotong University, Xi’an, Shaanxi Province, China; 2 Department of Neurosurgery, The First Affiliated Hospital of Henan University, Kaifeng, Henan Province, China; 3 Department of Neurology, The First Affiliated Hospital of Henan University, Kaifeng, Henan Province, China

**Keywords:** chemo-sensitization, glioblastoma, nectin-4, resveratrol, temozolomide

## Abstract

**Background:**

Temozolomide (TMZ) remains the first-line chemotherapeutic agent for glioblastoma (GBM), but its clinical efficacy is often limited by acquired resistance. This study aimed to investigate whether nectin-4 could serve as a target to sensitize GBM cells to TMZ and to determine whether resveratrol enhances TMZ efficacy through downregulation of nectin-4.

**Methods:**

Nectin-4 expression was evaluated in TMZ-treated GBM cell lines (U87, U251) and in an orthotopic GBM mouse model using qRT-PCR, Western blotting, and immunohistochemistry. Gain- and loss-of-function approaches were performed to assess the functional role of nectin-4 in GBM cell proliferation, tumor progression, and response to TMZ. The effects of resveratrol on nectin-4 expression and on the antitumor activity of TMZ were examined *in vitro* and in an intracranial orthotopic GBM model.

**Results:**

TMZ treatment significantly upregulated nectin-4 expression at both mRNA and protein levels in GBM cells and in tumor tissues from orthotopic mouse models. Nectin-4 overexpression promoted GBM cell proliferation *in vitro* and accelerated intracranial tumor progression *in vivo*, whereas nectin-4 knockdown enhanced the antitumor activity of TMZ. Resveratrol alone effectively reduced nectin-4 expression and blocked the TMZ-induced upregulation of nectin-4. Consistent with these molecular findings, the combination of resveratrol and TMZ synergistically suppressed GBM cell proliferation, inhibited intracranial tumor growth, and significantly prolonged survival of tumor-bearing mice compared with either agent alone.

**Conclusion:**

Targeting nectin-4 represents an effective strategy to overcome TMZ resistance in GBM. Resveratrol enhances TMZ efficacy at least in part through downregulating nectin-4, supporting its potential as a chemo-sensitizing adjunct to TMZ-based chemotherapy for GBM patients.

## 1 Introduction

Glioblastoma multiforme (GBM) is the most common and aggressive primary brain tumor, arising from neuroglial progenitor cells or neural stem cells in the central nervous system (Pouyan et al., 2025). Temozolomide (TMZ), an alkylating agent derived from dacarbazine, serves as the primary chemotherapeutic option for GBM (De Luca et al., 2026). However, the prognosis for GBM patients remains poor, with a median survival of only 15–18 months (Netto et al., 2026). Therefore, there is an urgent need to identify effective therapeutic targets that can enhance the responsiveness of glioma cells to TMZ.

Nectin cell adhesion protein 4 (Nectin-4), a transmembrane receptor encoded by the poliovirus receptor-related 4 (PVRL4) gene, plays essential roles in cellular adhesion, proliferation, differentiation, and signal transduction (Chatterjee et al., 2021; Nikanjam et al., 2025). Elevated expression of nectin-4 has been observed in various malignancies, including urothelial cancer, breast cancer, gastric cancer, and osteosarcoma (Heath and Rosenberg, 2021; Wali et al., 2025; Zhang et al., 2018; Liu et al., 2022). An antibody-drug conjugate (ADC) targeting nectin-4 has received approval for the treatment of locally advanced or metastatic urothelial cancer, underscoring its potential as a therapeutic target (Khosravanian et al., 2024). Notably, in colorectal cancer, nectin-4 overexpression has been shown to drive resistance to 5-fluorouracil (5-FU) through activation of the PI3K-AKT signaling cascade, and its knockdown effectively restores chemosensitivity (D et al., 2015). Although high nectin-4 expression is associated with unfavorable prognosis in glioma patients (Dekanic et al., 2023), whether it can serve as a sensitizing target to enhance TMZ efficacy in GBM remains to be elucidated.

Resveratrol, a natural polyphenol, has demonstrated broad antitumor activity in various cancers, including glioblastoma, through induction of apoptosis, cell cycle arrest, and inhibition of invasion (Ren et al., 2021; Khotchawan et al., 2026). Importantly, emerging evidence indicates that resveratrol sensitizes GBM cells to temozolomide and counteracts TMZ resistance (Wu et al., 2023; Liu et al., 2020). Notably, resveratrol has been shown to suppress nectin-4 expression and reverse chemoresistance in colorectal cancer (D et al., 2015). However, whether resveratrol enhances TMZ efficacy through nectin-4 downregulation in glioblastoma remains unknown.

Therefore, this study aimed to investigate whether resveratrol potentiates the antitumor activity of temozolomide in glioblastoma by suppressing nectin-4 expression, and to evaluate this combination strategy *in vitro* and in an orthotopic GBM model.

## 2 Materials and methods

### 2.1 Reagents and antibodies

Temozolomide (T2577) and resveratrol (R5010) were purchased from Sigma-Aldrich (Darmstadt, Germany). A rabbit polyclonal antibody against nectin-4 (ab235897) was purchased from Abcam (Cambridge, MA, USA). Mouse anti-β-actin (TA-09), horseradish peroxidase (HRP)-conjugated goat anti-rabbit IgG (ZB-2301) and goat anti-mouse IgG (ZB-2305) were purchased from Zhongshan Golden Bridge Biotechnology (Beijing, China).

### 2.2 Cell culture

Glioblastoma cell lines (U87MG, U251, and GL261) were acquired from the Cell Bank of the Chinese Academy of Science (Shanghai, China). Cells were maintained in Dulbecco’s modified Eagle’s medium (Gibco, Carlsbad, CA, USA) supplemented with 10% heat-inactivated fetal bovine serum (PAN-Biotech, Aidenbach, Germany), 100 U/mL penicillin, and 100 μg/mL streptomycin (Solarbio, Beijing, China), under a humidified atmosphere of 5% CO_2_ at 37 °C in a HERAcell 150i incubator (Thermo Fisher Scientific, Waltham, MA, USA). All cell culture procedures were performed in a Class II biological safety cabinet (ESCO, Singapore).

### 2.3 Plasmid construction and lentivirus production

Lentiviral vectors were employed to achieve stable overexpression or knockdown of nectin-4. For ectopic expression, the full-length Nectin-4 coding sequence was synthesized and inserted into the pCDH-CMV-MCS-EF1-CopGFP-T2A-Puro vector. For silencing, a short hairpin RNA (shRNA) targeting nectin-4 was cloned into the hU6-MCS-CBh-gcGFP-IRES-puromycin plasmid. Lentiviral particles were generated by co-transfecting HEK-293 T cells with the transfer vector along with the packaging plasmid psPAX2 and the envelope plasmid pMD2.G. Viral supernatants were filtered using 0.45 μm pore-size membranes and concentrated with Vivaspin® 20 centrifugal concentrators (Sartorius, Göttingen, Germany). Target cells were then transduced with the concentrated lentivirus, followed by selection with 1.5 μg/mL puromycin (Sigma, St. Louis, MO, USA) for 2 weeks.

### 2.4 Cell viability assay

Glioblastoma cells were seeded into 96-well plates (Corning, Corning, NY, USA) at 1,000 cells per well and cultured for various durations. Subsequently, 10 μL of MTT solution (5 mg/mL, Solarbio, Beijing, China) was added to each well, and plates were incubated for 4 h at 37 °C. The resulting formazan crystals were dissolved in 100 μL of dimethyl sulfoxide (DMSO). Absorbance was measured at 570 nm using a microplate reader (Thermo Scientific, Rockford, IL, USA).

### 2.5 Transcriptomic data re-analysis

Publicly available RNA-seq datasets were retrieved from the Gene Expression Omnibus (GEO) database under accession numbers GSE275875 (GBM tumorsphere cells treated with TMZ) and GSE296486 (TMZ-treated subcutaneous tumor tissues). Normalized expression values for nectin-4 (log_2_ TPM +1) were extracted from the processed data provided in each dataset. Statistical comparisons between TMZ-treated and control groups were performed using unpaired Student’s t-test. A p-value <0.05 was considered statistically significant. All data processing and visualization were conducted in R (version 4.2.0).

### 2.6 Real-time quantitative PCR (qPCR)

Total RNA was isolated from cultured cells and tumor tissues using TRIzol reagent (Invitrogen, Carlsbad, CA, USA) according to the manufacturer’s protocol. RNA concentration and purity were determined by spectrophotometry (NanoDrop, Thermo Fisher Scientific). Reverse transcription was performed with PrimeScript RT Master Mix (Takara, Tokyo, Japan). qPCR was conducted using 2× SYBR Green PCR Master Mix (Promega, Madison, WI, USA) on an ABI 7500 real-time PCR system (Applied Biosystems, Foster City, CA, USA). Relative gene expression was calculated using the 2^−^ΔΔCt method. The primer sequences were as follows: Human nectin-4: forward 5′- TCC AGG ACC AAA GGA TCA CC -3′, reverse 5′- AGC CGT GTC CAG TTG TAT GA -3′; Human GAPDH: forward 5′- GAC ACC CAC TCC ACC TTT -3′, reverse 5′- TTG CTG TAG CCA AAT TCG TTGT -3′; mouse nectin-4: forward 5′- GGC ATC GTT TAC AGG CCA AT -3′, reverse 5′- AGC ACC ACT GTC ACT ACG TCA GA -3′; mouse GAPDH: forward 5′- ACC CAG AAG ACT GTG GAT GG -3′, reverse 5′- CAC ATT GGG GGT AGG AAC AC -3′.

### 2.7 Western blotting

For cultured cells, protein lysates were prepared using RIPA lysis buffer (Solarbio, Beijing, China) supplemented with protease inhibitors (Roche, Basel, Switzerland). Cells were lysed on ice for 30 min and centrifuged at 12,000 × g for 15 min at 4 °C, and the supernatants were collected. For tumor tissues, approximately 50 mg of snap-frozen tissue was homogenized in RIPA lysis buffer supplemented with protease inhibitors using a tissue homogenizer, and the homogenates were centrifuged at 12,000 × g for 20 min at 4 °C to collect the supernatants. Protein concentrations were determined with a BCA assay kit (Pierce, Rockford, IL, USA). Equal amounts of protein were separated by 10% SDS-polyacrylamide gel electrophoresis (SDS-PAGE) and transferred onto polyvinylidene difluoride (PVDF) membranes. After blocking with 5% non-fat milk for 1 h at room temperature, membranes were incubated overnight at 4 °C with primary antibodies against nectin-4 (1:1,000) and β-actin (1:2000), followed by incubation with horseradish peroxidase (HRP)-conjugated secondary antibodies (1:1,000) for 1 h at room temperature. Signal detection was carried out using enhanced chemiluminescence (ECL) substrate (BeyoECL Plus, Beyotime Biotechnology, Shanghai, China) on a FluorChem E Imager System (Protein Simple, San Jose, CA, USA). Densitometric analysis was performed using Quantity One software (Bio-Rad, Hercules, CA, USA).

### 2.8 Animal studies

Male C57BL/6 J Nifdc mice (5–6 weeks old) were obtained from Beijing Weitong Lihua Animal Co. (Beijing, China) and allowed to acclimatize for 1 week prior to experiments. The animal study was approved by Animal Ethics Committee of Henan University. The study was conducted in accordance with the local legislation and institutional requirements.

For orthotopic tumor implantation, mice were anesthetized using isoflurane and positioned in a stereotaxic apparatus (RWD, Shenzhen, China). After a midline scalp incision, a burr hole was drilled at 2.0 mm lateral and 0.7 mm posterior to the bregma using a handheld drill. Tumor cells (3 × 10^5^ in 5 μL PBS) were injected through a 26-gauge needle attached to a 10-μL Hamilton microsyringe (Hamilton, Reno, NV, USA) at a depth of 3.0 mm below the dura. The needle was withdrawn slowly 3 min post-injection to prevent reflux. The hole was sealed with bone wax and the incision was sutured. To evaluate the effect of TMZ on nectin-4 expression, luciferase-labeled GL261 cells (3 × 10^5^ cells/mouse) were intracranially injected into mice. One week later, by which time tumor engraftment had been established as confirmed by bioluminescence imaging, animals received intraperitoneal injections of either 25 mg/kg TMZ or vehicle (5% DMSO, 30% PEG300, 65% ddH_2_O) once daily for 2 weeks. Tumor progression was monitored weekly using the IVIS Spectrum imaging system. At the end of the treatment, mice were euthanized by cervical dislocation, and brain tissues were harvested for analysis of nectin-4 expression.

To investigate the tumorigenic role of nectin-4, GL261 cells stably overexpressing nectin-4 or control cells (3 × 10^5^ cells/mouse) were implanted into the mouse brain. Tumor growth was assessed after 2 weeks using bioluminescence imaging. To examine whether nectin-4 knockdown enhances TMZ efficacy, GL261 cells with stable nectin-4 shRNA or control shRNA were injected intracranially. After 1 week, mice were treated with 25 mg/kg TMZ or vehicle daily for 2 weeks, and tumor burden was monitored by imaging.

For combination therapy evaluation, an orthotopic GBM model was established by injecting 3 × 10^5^ luciferase-labeled GL261 cells into the brain. One week after implantation, mice received daily intraperitoneal injections of vehicle, TMZ (25 mg/kg), resveratrol (20 mg/kg), or a TMZ/resveratrol combination for 2 weeks. All drugs were administered in a volume of 10 mL/kg body weight. The dose of resveratrol was chosen based on published study demonstrating its antitumor efficacy at this dosage (Zhong et al., 2019). Animal survival was recorded, and tumor growth was monitored using the IVIS system.

For bioluminescence imaging, mice were injected intraperitoneally with D-luciferin (150 mg/kg; PerkinElmer, Waltham, MA, USA) and anesthetized with isoflurane. Regions of interest (ROIs) of identical size were drawn around the tumor area of each mouse, and bioluminescence signals were quantified as radiance (photons/sec/cm^2^/sr). The same ROI size and position were applied consistently across all mice. Statistical comparisons between groups were performed using the absolute radiance values at each time point, and all imaging data are presented as mean ± SEM.

### 2.9 Immunohistochemistry (IHC)

Mouse brain tissues were fixed in paraffin and sectioned into 4 μm thick slices, which were mounted onto polylysine-coated slides. After deparaffinization and rehydration through graded alcohols, endogenous peroxidase activity was blocked with 3% H_2_O_2_ for 20 min at room temperature. Antigen retrieval was performed using 0.1% citric acid buffer at 95 °C for 30 min. Sections were then incubated overnight at 4 °C with primary antibodies against nectin-4, followed by incubation with a streptavidin-conjugated HRP secondary antibody for 30 min. Staining was visualized using diaminobenzidine (DAB). Two independent pathologists, blinded to the experimental groups, evaluated the staining. For each section, five representative fields at ×200 magnification were randomly selected and examined. The staining results were assessed using a semi-quantitative scoring system based on two parameters: the proportion of positively stained tumor cells (percentage score) and the staining intensity (intensity score). The percentage score reflects the estimated fraction of tumor cells exhibiting positive immunoreactivity, graded on a 6-tier scale: 0 (no positive cells), 1 (<1%), 2 (2%–10%), 3 (11%–30%), 4 (31%–70%), and 5 (71%–100%). The intensity score reflects the strength of the immunostaining signal, graded on a 4-tier scale: 0 (negative, no detectable staining), 1 (weak, barely visible staining), 2 (moderate, clearly distinguishable staining), and 3 (strong, intense dark staining). The final IHC score for each section was calculated by adding the percentage score to the intensity score, yielding a composite value ranging from 0 to 8.

### 2.10 Statistical analysis

All data are presented as mean ± standard error of the mean (SEM). Statistical analyses were conducted using GraphPad Prism 8.0 software (GraphPad Software, Inc., San Diego, CA, USA). Comparisons between two groups were performed using unpaired Student’s t-test. For multiple group comparisons, one-way or two-way analysis of variance (ANOVA) was applied. A p-value <0.05 was considered statistically significant.

## 3 Results

### 3.1 Temozolomide promotes nectin-4 expression in glioblastoma

**FIGURE 1:**
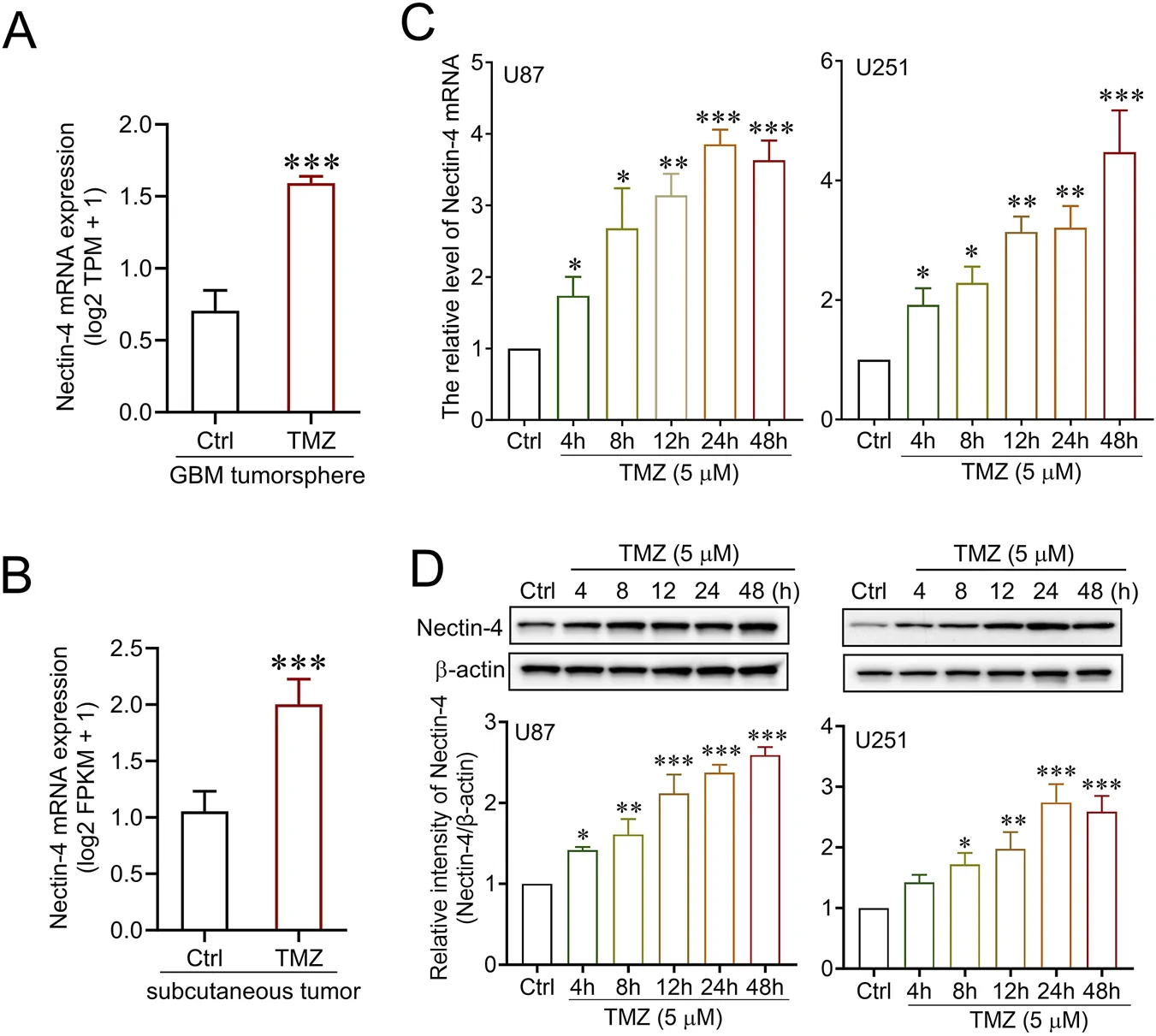
TMZ induces Nectin-4 expression in GBM cells. **(A)** Nectin-4 mRNA expression in GBM tumorsphere cells treated with vehicle or TMZ (re-analyzed from GEO dataset GSE275875). ***p < 0.001, unpaired Student’s t-test. **(B)** Nectin-4 expression in TMZ-treated subcutaneous tumor tissues (re-analyzed from GEO dataset GSE296486). *p < 0.05, unpaired Student’s t-test. **(C)** qRT-PCR analysis of nectin-4 mRNA levels in U87 and U251 cells treated with 5 μM TMZ or vehicle for the indicated time periods (4, 8, 12, 24, and 48 h). N = 3 independent experiments per group, *p < 0.05, **p < 0.01, ***p < 0.001 vs*.* control group, One-way ANOVA followed by Tukey’s multiple comparisons test. **(D)** Representative Western blots (upper panels) and densitometric quantification (lower panels) of nectin-4 protein expression in U87 and U251 cells treated with 5 μM TMZ or vehicle for the indicated time periods (4, 8, 12, 24, and 48 h). β-Actin served as a loading control. N = 3 independent experiments per group, *p < 0.05, **p < 0.01, ***p < 0.001 vs*.* control, one-way ANOVA followed by Tukey’s multiple comparisons test.

To explore potential molecular targets for improving the therapeutic response to temozolomide (TMZ), we re-analyzed publicly available transcriptomic datasets from the Gene Expression Omnibus (GEO) database. In GBM tumorsphere cells treated with TMZ (GSE275875), nectin-4 expression was significantly upregulated following TMZ exposure compared with untreated controls (Figure 1A). To validate this finding in an independent dataset, we further analyzed another publicly available RNA-seq dataset (GSE296486) derived from TMZ-treated subcutaneous tumor tissues, which also confirmed the upregulation of nectin-4 mRNA upon TMZ treatment (Figure 1B). This upregulation was further validated in two GBM cell lines, U87 and U251, where exposure to 5 μM TMZ consistently enhanced both nectin-4 mRNA and protein expression (Figures 1C,D).

**FIGURE 2:**
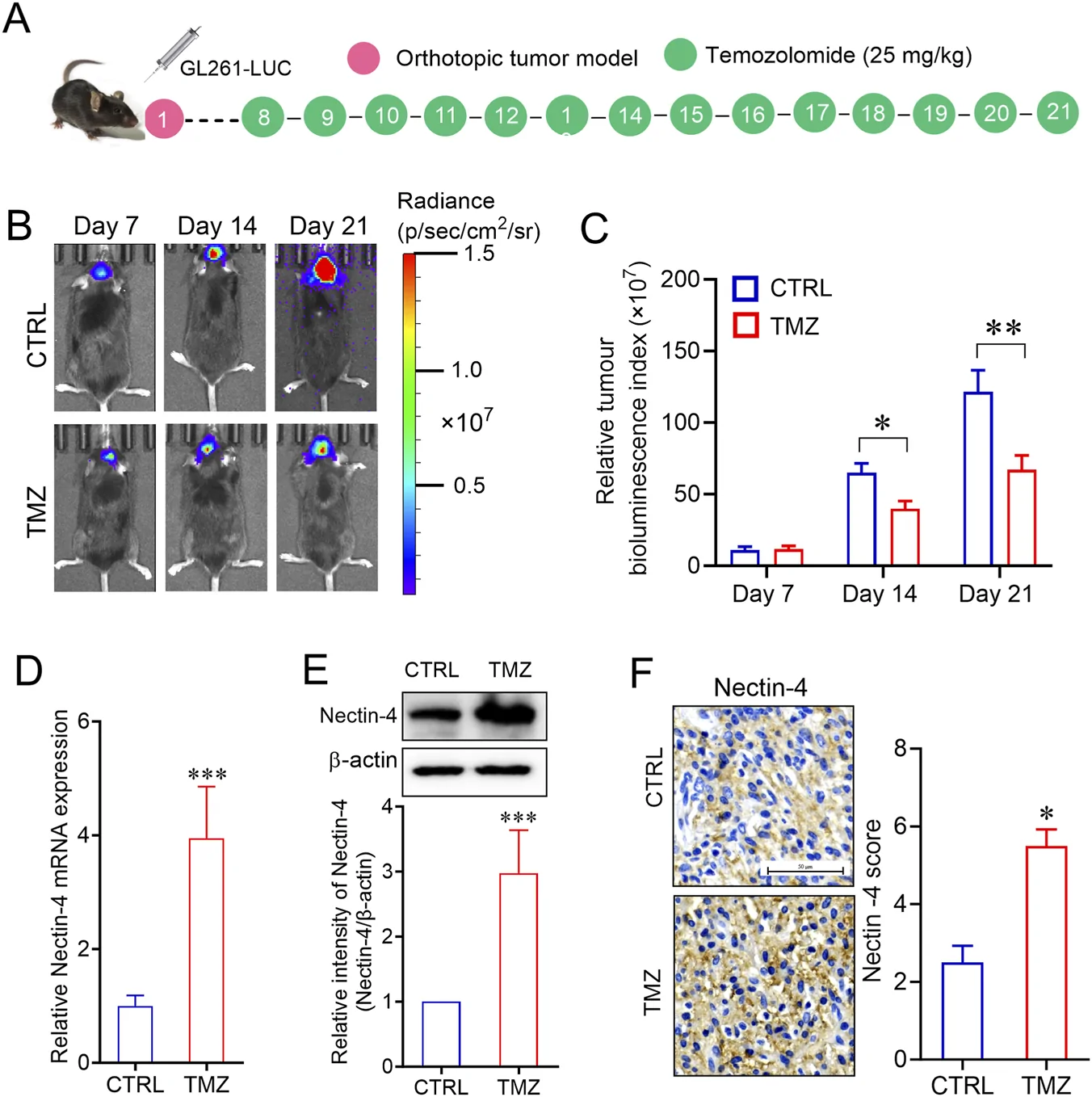
TMZ treatment upregulates Nectin-4 expression *in vivo*. **(A)** Schematic of the orthotopic GBM model and TMZ treatment regimen. **(B)** Representative bioluminescence images of vehicle- and TMZ-treated mice. **(C)** Quantification of bioluminescence signal intensity (radiance) over time. N = 6 mice per group, *p < 0.05, **p < 0.01, two-way ANOVA followed by Bonferroni’s *post hoc* test. **(D)** qRT-PCR analysis of Nectin-4 mRNA levels in resected brain tumor tissues from vehicle- and TMZ-treated mice. N = 3 mice per group, ***p < 0.001 vs*.* vehicle group, unpaired Student’s t-test. **(E)** Representative Western blots (upper panels) and densitometric quantification (lower panels) of Nectin-4 protein expression in tumor tissues from the indicated groups. N = 3 mice per group. ***p < 0.001 vs. vehicle group, unpaired Student’s t-test. **(F)** Immunohistochemical staining confirming elevated Nectin-4 protein abundance in TMZ-treated tumors. N = 6 mice per group, *p < 0.05 vs*.* vehicle group, unpaired Student’s t-test.

To investigate whether TMZ exerts a similar effect *in vivo*, an orthotopic GBM model was established by intracranially implanting luciferase-labeled GL261 cells into mice. After 1 week of tumor engraftment, animals received daily intraperitoneal injections of either 25 mg/kg TMZ or vehicle control for two consecutive weeks (Figure 2A). Bioluminescence imaging demonstrated that TMZ administration suppressed intracranial tumor progression (Figures 2B,C). Concurrently, qRT-PCR and Western blot analyses of resected brain tumor tissues revealed that TMZ treatment significantly elevated nectin-4 mRNA and protein levels compared with the vehicle group (Figures 2D,E). Immunohistochemical staining further confirmed increased Nectin-4 protein abundance in TMZ-treated tumor specimens (Figure 2F).

### 3.2 Nectin-4 serves as a potential sensitizing target for TMZ in glioblastoma

**FIGURE 3:**
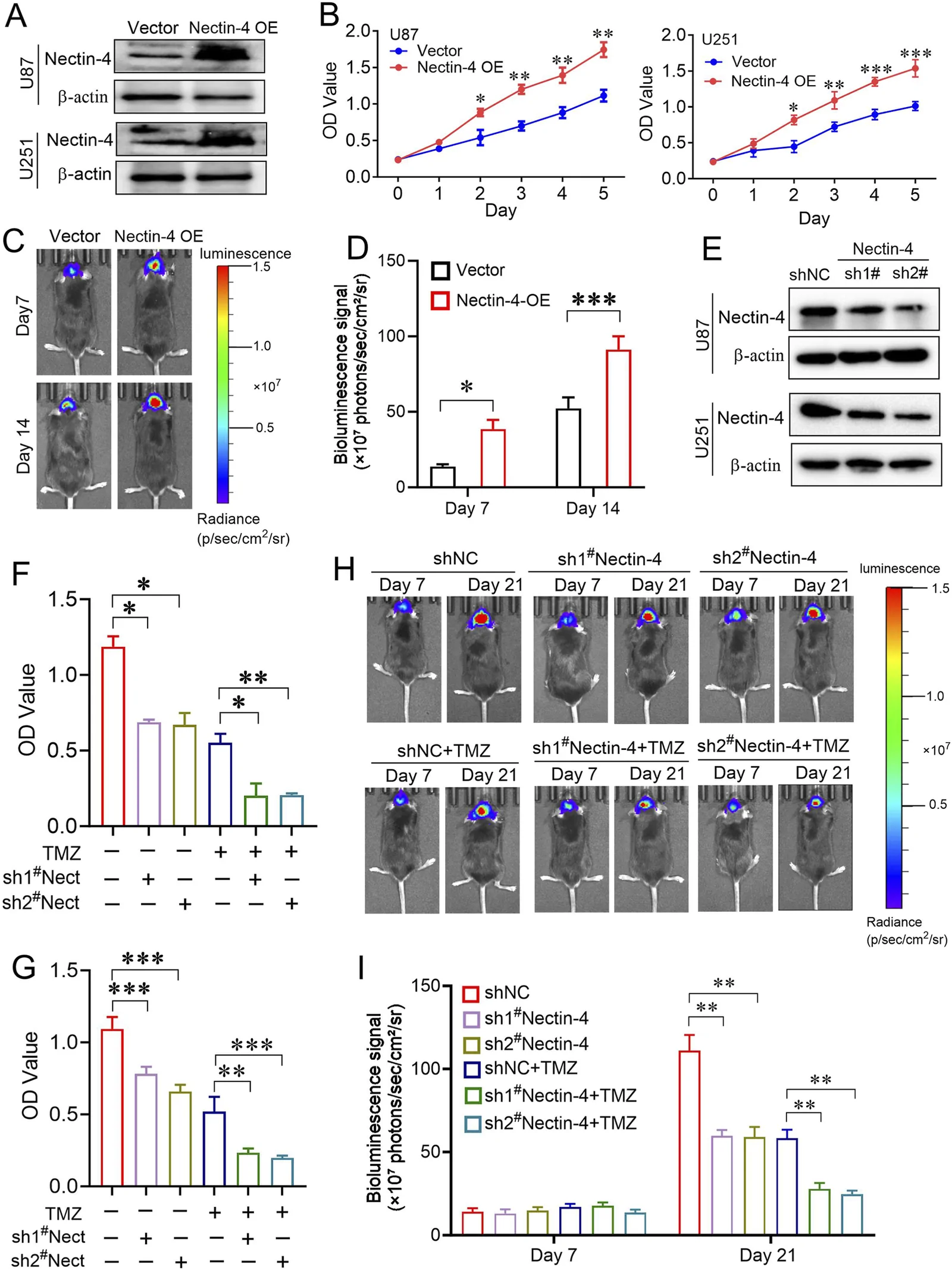
Nectin-4 promotes GBM progression and serves as a sensitizing target for TMZ. **(A)** Validation of nectin-4 overexpression in U87 and U251 cells by Western blot. **(B)** MTT assay evaluating cell proliferation in U87 and U251 cells with nectin-4 overexpression or vector control over the indicated time course. N = 5 independent experiments, *p < 0.01, **p < 0.01, ***p < 0.001 vs*.* Vector group, two-way ANOVA followed by Bonferroni’s *post hoc* test. **(C)** Representative bioluminescence images of orthotopic GBM models established with luciferase-labeled GL261 cells overexpressing Nectin-4 or control vector (Vector). **(D)** Quantification of bioluminescence signal intensity. N = 6 mice per group, *p < 0.01, ***p < 0.001, two-way ANOVA followed by Bonferroni’s *post hoc* test. **(E)** Validation of nectin-4 knockdown in U87 and U251 cells by Western blot. **(F,G)** MTT assay evaluating the growth inhibitory effects of TMZ in U87 **(F)** and U251 **(G)** cells with nectin-4 knockdown (shNect). N = 3 independent experiments, *p < 0.01, **p < 0.01, ***p < 0.001, two-way ANOVA followed by Tukey’s multiple comparisons test. **(H)** Representative bioluminescence images of orthotopic GBM models with Nectin-4 knockdown followed by vehicle or TMZ treatment. **(I)** Quantification of bioluminescence signal intensity. N = 6 mice per group, **p < 0.01, two-way ANOVA followed by Bonferroni’s *post hoc* test.

To investigate the functional role of Nectin-4 in modulating the therapeutic response of glioblastoma to temozolomide (TMZ), we first established U87 and U251 cell lines with stable nectin-4 overexpression via lentiviral transduction. The efficiency of nectin-4 overexpression was confirmed at protein level (Figure 3A). Subsequently, MTT assays were performed to evaluate the impact of nectin-4 upregulation on cell proliferation. The results demonstrated that cells with ectopic nectin-4 expression exhibited a significantly higher proliferative capacity compared with vector control cells over the observed time course (Figure 3B).

To further validate these findings in an *in vivo* setting, an intracranial orthotopic GBM model was established using luciferase-labeled GL261 cells engineered to stably overexpress nectin-4. Tumor growth was monitored longitudinally by bioluminescence imaging. As shown in Figures 3C,D, nectin-4 overexpression dramatically accelerated intracranial tumor progression relative to the control group, as evidenced by both increased bioluminescent signal intensity.

To determine whether nectin-4 knockdown directly sensitizes GBM cells to TMZ *in vitro*, we generated U87 and U251 cells stably expressing nectin-4-specific shRNA (Figure 3E). Cells were then treated with 5 μM TMZ or vehicle for 48 h, and cell viability was assessed by MTT assay. As shown in Figures 3F,G, TMZ treatment significantly reduced cell viability in both U87 and U251 cells; importantly, nectin-4 knockdown markedly enhanced the growth inhibitory effect of TMZ.

To further validate these findings *in vivo*, we generated GL261 cells stably expressing nectin-4-specific shRNA. Following 1 week of tumor engraftment, mice bearing either control or nectin-4 knockdown tumors were subjected to daily intraperitoneal injections of 25 mg/kg TMZ or vehicle for two consecutive weeks. Bioluminescence imaging revealed that TMZ treatment alone partially suppressed tumor growth; notably, the combination of nectin-4 knockdown and TMZ administration resulted in a substantially greater reduction in tumor burden compared with TMZ treatment alone (Figures 3H,I). Collectively, these findings strongly suggest that nectin-4 represents a promising and actionable target for improving glioblastoma sensitivity to TMZ-based chemotherapy.

### 3.3 Resveratrol enhances the anti-tumor activity of TMZ by inhibiting Nectin-4 expression in GBM

**FIGURE 4:**
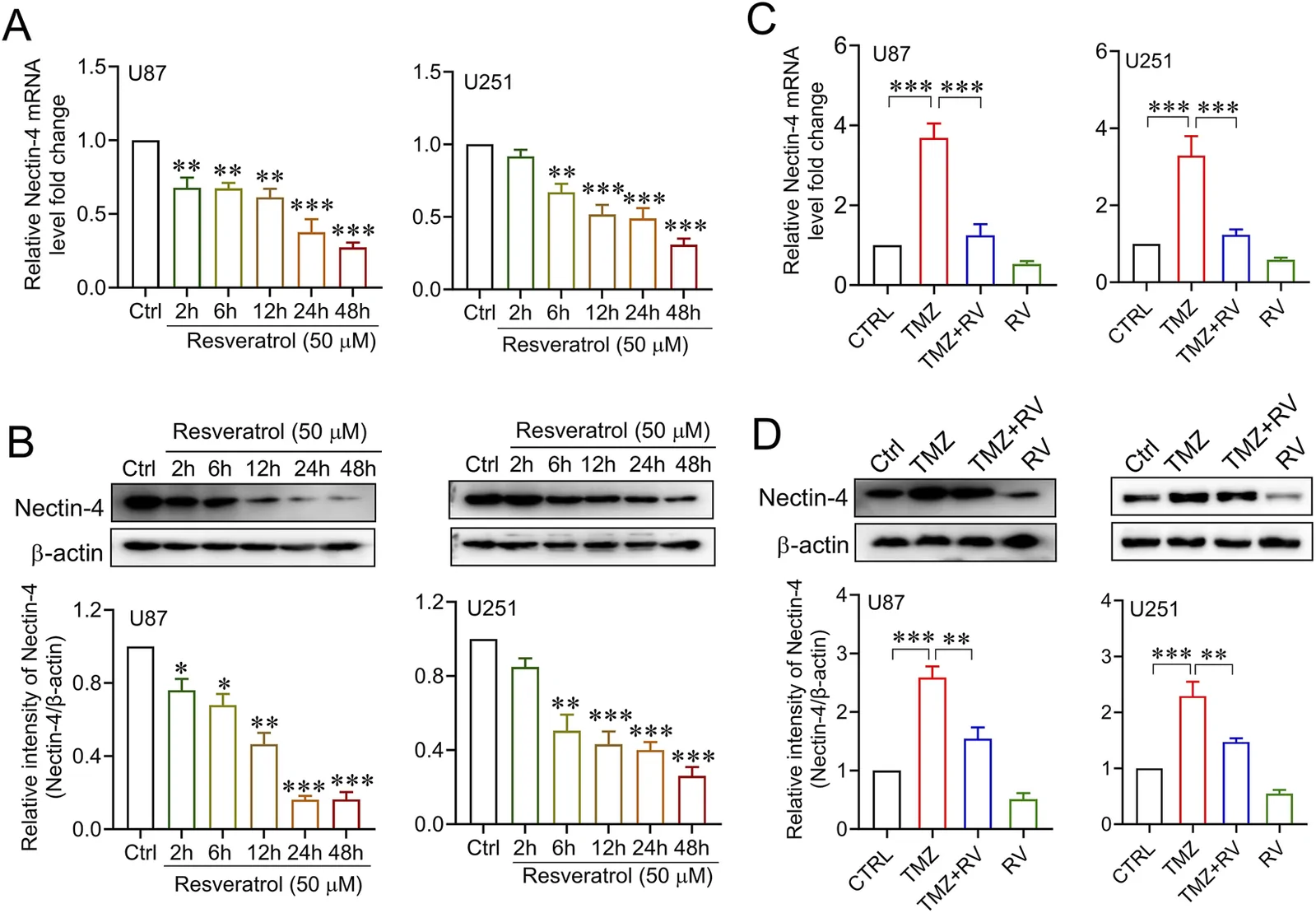
Resveratrol suppresses nectin-4 expression and counteracts TMZ induced upregulation in GBM cells. **(A,B)** Resveratrol inhibited nectin-4 mRNA **(A)** and protein **(B)** expression in U87 and U251 cells. Cells were treated with resveratrol at 50 μM for the indicated time periods (2, 6, 12, 24, and 48 h). N = 4 independent experiments per group, *p < 0.05, **p < 0.01, ***p < 0.001 vs. control group, one-way ANOVA followed by Tukey’s multiple comparisons test. **(C,D)** Resveratrol blocked TMZ-induced nectin-4 mRNA **(C)** and protein **(D)** expression in U87 and U251 cells. N = 4 independent experiments per group, **p < 0.01, ***p < 0.001, one-way ANOVA followed by Tukey’s multiple comparisons test.

Given that resveratrol has been shown to suppress nectin-4 expression and enhance chemosensitivity in colon cancer cells (D et al., 2015), we investigated whether resveratrol could exert a similar effect by downregulating nectin-4 to potentiate the anti-GBM activity of temozolomide. To test this possibility, U87 and U251 cells were treated with resveratrol at 50 μM, a concentration previously shown to exert biological effects in glioma cells (Öztürk et al., 2019). Real time PCR and Western blot analysis revealed that resveratrol significantly reduced both mRNA and protein levels of nectin-4 (Figures 4A,B). We next examined whether resveratrol could counteract the elevation of nectin-4 induced by temozolomide. Cells were co-incubated with resveratrol and temozolomide, and the results showed that resveratrol significantly attenuated the temozolomide mediated increase in nectin-4 expression at both transcriptional and translational levels (Figures 4C,D). These findings suggest that resveratrol may potentiate the antitumor effect of temozolomide at least in part through suppressing nectin-4 in glioblastoma cells.

**FIGURE 5:**
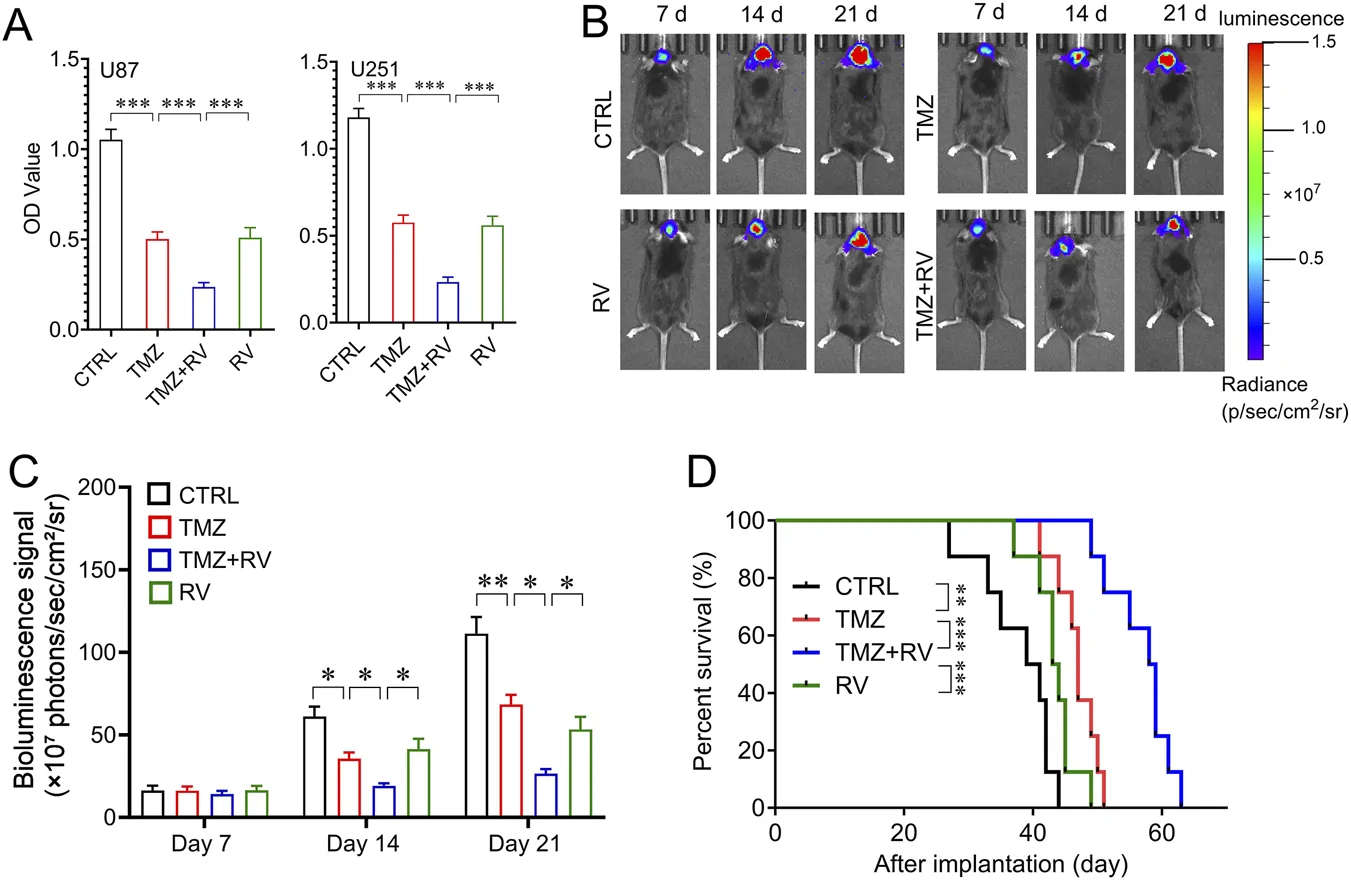
Resveratrol synergizes with TMZ to suppress GBM growth *in vitro* and *in vivo.* **(A)** MTT assay evaluating the antiproliferative effects of resveratrol, TMZ, and their combination in U87 and U251 GBM cells. N = 5 independent experiments, ***p < 0.001, one-way ANOVA followed by Tukey’s multiple comparisons test. **(B,C)** Representative bioluminescence images of mice from each treatment group **(B)** and quantification of bioluminescence signal intensity **(C)**, n = 8 mice per group, *p < 0.05, **p < 0.01, two-way ANOVA followed by Bonferroni’s *post hoc* test. **(D)** Kaplan-Meier survival curves of tumor-bearing mice from each treatment group, n = 8 mice per group. **p < 0.01, ***p < 0.001, Statistical significance was determined by the log-rank test.

We next investigated whether resveratrol could enhance the anti-GBM activity of TMZ when used in combination. In both U87 and U251 cell lines, the combination of resveratrol and TMZ produced substantially lower OD values than either agent alone (Figure 5A). To further evaluate this effect *in vivo*, we used an orthotopic GBM model. Bioluminescence imaging showed that tumor growth was suppressed in mice receiving the combination treatment, compared with the control group or either monotherapy (Figures 5B,C). Accordingly, Kaplan-Meier survival analysis demonstrated that the combination of resveratrol and TMZ significantly prolonged the survival of tumor-bearing mice relative to resveratrol or TMZ alone in the orthotopic model (Figure 5D). Collectively, these results indicate that resveratrol synergizes with TMZ to suppress tumor growth and extend survival in GBM models, supporting its potential as a chemo-sensitizing partner in GBM treatment.

## 4 Discussion

Temozolomide (TMZ) has become a mainstay in the management of glioblastoma (GBM), owing to its potent antitumor activity and favorable ability to penetrate the blood-brain barrier (BBB) (Jezierzanski et al., 2024). Nevertheless, accumulating evidence indicates that its clinical efficacy is frequently compromised by hyperactivation of DNA repair mechanisms, enhanced drug efflux, and aberrant apoptotic signaling (Zhang et al., 2021; Yang et al., 2022; Wu et al., 2020; Hanif et al., 2018). Our study provides novel insights demonstrating that nectin-4 upregulation attenuates the therapeutic response to TMZ, whereas resveratrol enhances TMZ antitumor efficacy by suppressing nectin-4 axis in GBM.

Nectin-4 is a transmembrane cell adhesion molecule belonging to the nectin family of immunoglobulin-like proteins (Nikanjam et al., 2025). Recently, Nectin-4 has garnered significant attention as a tumor-associated antigen due to its aberrant overexpression in a wide range of malignancies, including urothelial carcinoma, breast cancer, pancreatic cancer, lung cancer, ovarian cancer, and gastric cancer (Chatterjee et al., 2021; Takano et al., 2009; Rogmans et al., 2022). Notably, this overexpression has been successfully exploited for therapeutic purposes, as demonstrated by the FDA approval of enfortumab vedotin, an antibody-drug conjugate targeting nectin-4, for the treatment of locally advanced or metastatic urothelial carcinoma (Klümper et al., 2023; Aggen et al., 2023). Within the context of gliomas, previous studies have reported that elevated nectin-4 expression correlates with shorter overall survival in patients with grade II/III gliomas, suggesting its potential utility as an independent prognostic indicator for this patient population (Dekanic et al., 2023). However, the functional role of nectin-4 specifically in glioblastoma has remained largely unexplored, and whether this prognostic association reflects a direct causal contribution to tumor aggressiveness or merely an epiphenomenon has been unclear. Beyond confirming previous prognostic observations, our findings provide direct functional evidence that nectin-4 actively promotes GBM proliferation and progression, as demonstrated by both gain-of-function and loss-of-function experiments *in vitro* and *in vivo*. This establishes nectin-4 as not just a passive prognostic marker but an actionable therapeutic target in GBM. Furthermore, we observed that TMZ treatment itself upregulates nectin-4 expression, raising the possibility that chemotherapy-induced nectin-4 elevation may serve as an adaptive resistance mechanism that limits the durability of TMZ response.

Accumulating evidence has implicated nectin-4 in the malignant progression of multiple cancer types. For instance, nectin-4 has been shown to enhance tumor cell proliferation, migration, and invasion by activating the PI3K/AKT/NF-κB signaling axis in osteosarcoma, and by engaging the PI3K/AKT pathway to upregulate Rac1 in gallbladder cancer (Liu et al., 2022; Zhang et al., 2016). Beyond its direct effects on cancer cells, nectin-4 also contributes to tumor aggressiveness through stem cell-related mechanisms. In breast cancer, nectin-4 serves as a marker of cancer stem cells and promotes self-renewal via WNT/β-catenin signaling downstream of the PI3K/AKT axis (Siddharth et al., 2017). Furthermore, in pancreatic ductal adenocarcinoma, nectin-4 expression by tumor cells correlates with low immune infiltration and directly suppresses T cell effector cytokine production, suggesting an immunomodulatory role (Heiduk et al., 2026). Taken together, these findings lead us to propose that similar molecular mechanisms, particularly PI3K/AKT-driven signaling cascades, cancer stem cell maintenance, and immune evasion, may underlie nectin-4-induced malignant progression in glioblastoma.

Multiple studies have established resveratrol as a chemosensitizer that enhances temozolomide efficacy in glioblastoma through various mechanisms. These include inhibition of O6 methylguanine DNA methyltransferase (MGMT), suppression of ROS/ERK mediated protective autophagy, downregulation of P glycoprotein via MAPK/ERK pathway blockade, and attenuation of Wnt/β catenin signaling (Wu et al., 2023; Rezaie et al., 2025; Lin et al., 2012; Zhang et al., 2025). Extending these observations, our study identifies nectin-4 as a previously unrecognized target of resveratrol in GBM. We demonstrated that resveratrol alone suppressed nectin-4 expression at both mRNA and protein levels in GBM cells. More importantly, resveratrol effectively counteracted the TMZ induced upregulation of nectin-4, a phenomenon we found to be associated with TMZ treatment. This finding is particularly relevant as TMZ induced nectin-4 elevation may represent an adaptive resistance mechanism, and the ability of resveratrol to block this elevation parallels its known effect on TMZ induced MGMT upregulation. Regarding the mechanism by which resveratrol suppresses nectin-4, we speculate that this effect may occur through inhibition of the PI3K/AKT pathway, as resveratrol has been shown to downregulate nectin-4 via PI3K-AKT disruption in colorectal cancer (D et al., 2015). Given that resveratrol can cross the blood brain barrier and has a favorable safety profile, our findings support its further evaluation as an adjunct to TMZ based chemotherapy for GBM patients.

Notably, although the combination of TMZ and resveratrol did not suppress nectin-4 expression more profoundly than TMZ alone, it exhibited superior antitumor efficacy *in vivo*. This is because TMZ primarily kills tumor cells through DNA damage, while nectin-4 upregulation, induced by TMZ, serves as a resistance mechanism that partially counteracts this effect. Resveratrol, by suppressing nectin-4, removes this resistance-associated brake, thereby allowing TMZ to exert its full cytotoxic activity. Thus, the enhanced efficacy of the combination reflects the net benefit of relieving nectin-4-mediated resistance, rather than additive suppression of nectin-4 itself.

However, several aspects remain to be addressed. First, our study does not exclude the involvement of other known TMZ resistance mediators, including DKK1 and GDF15, both of which have previously been associated with chemoresistance in glioma (Zhang and Song, 2025; Zhang et al., 2024). Second, our gain-of-function experiments primarily established nectin-4 as a pro-proliferative factor in GBM, whereas a direct assessment of whether nectin-4 overexpression reduces TMZ sensitivity was not performed. Nevertheless, several lines of evidence support that nectin-4 contributes to TMZ resistance beyond its growth-promoting effect: TMZ itself upregulates nectin-4 as an adaptive response to chemotherapy, and nectin-4 knockdown sufficiently resensitizes GBM to TMZ *in vivo*. Third, and most importantly, while we have proposed that resveratrol suppresses nectin-4 through PI3K/AKT pathway inhibition based on published evidence in colorectal cancer (D et al., 2015), we have not provided direct mechanistic evidence in our GBM models. Future investigations examining AKT phosphorylation status following resveratrol treatment, as well as the use of specific pathway inhibitors, are warranted to definitively establish this regulatory axis. Additionally, it remains unclear whether nectin-4 knockdown enhances TMZ efficacy through reduced proliferation, increased apoptosis, improved immune infiltration, or a combination of these processes.

Despite these limitations, our study establishes nectin-4 as an actionable target to overcome TMZ resistance in GBM, and identifies resveratrol as a chemosensitizing agent that suppresses nectin-4 expression and counteracts TMZ-induced nectin-4 upregulation. Given that resveratrol can cross the blood-brain barrier and has a favorable safety profile, our findings provide a rationale for further evaluation of resveratrol as an adjunct to TMZ-based chemotherapy for GBM patients.

## Data Availability

The raw data supporting the conclusions of this article will be made available by the authors upon reasonable request, without undue reservation.
